# DRUM: Inference of Disease-Associated m^6^A RNA Methylation Sites From a Multi-Layer Heterogeneous Network

**DOI:** 10.3389/fgene.2019.00266

**Published:** 2019-04-03

**Authors:** Yujiao Tang, Kunqi Chen, Xiangyu Wu, Zhen Wei, Song-Yao Zhang, Bowen Song, Shao-Wu Zhang, Yufei Huang, Jia Meng

**Affiliations:** ^1^Department of Biological Sciences, Research Center for Precision Medicine, Xi'an Jiaotong-Liverpool University, Suzhou, China; ^2^Institute of Integrative Biology, University of Liverpool, Liverpool, United Kingdom; ^3^Institute of & Chronic Disease, University of Liverpool, Liverpool, United Kingdom; ^4^Key Laboratory of Information Fusion Technology of Ministry of Education, School of Automation, Northwestern Polytechnical University, Xi'an, China; ^5^Department of Epidemiology and Biostatistics, University of Texas Health San Antonio, San Antonio, TX, United States; ^6^Department of Electrical and Computer Engineering, University of Texas at San Antonio, San Antonio, TX, United States

**Keywords:** disease, RWR, random walk with restart, m6A modification, Co-expression, network analysis

## Abstract

Recent studies have revealed that the RNA *N*^6^-methyladenosine (m^6^A) modification plays a critical role in a variety of biological processes and associated with multiple diseases including cancers. Till this day, transcriptome-wide m^6^A RNA methylation sites have been identified by high-throughput sequencing technique combined with computational methods, and the information is publicly available in a few bioinformatics databases; however, the association between individual m^6^A sites and various diseases are still largely unknown. There are yet computational approaches developed for investigating potential association between individual m^6^A sites and diseases, which represents a major challenge in the epitranscriptome analysis. Thus, to infer the disease-related m^6^A sites, we implemented a novel multi-layer heterogeneous network-based approach, which incorporates the associations among diseases, genes and m^6^A RNA methylation sites from gene expression, RNA methylation and disease similarities data with the Random Walk with Restart (RWR) algorithm. To evaluate the performance of the proposed approach, a ten-fold cross validation is performed, in which our approach achieved a reasonable good performance (overall AUC: 0.827, average AUC 0.867), higher than a hypergeometric test-based approach (overall AUC: 0.7333 and average AUC: 0.723) and a random predictor (overall AUC: 0.550 and average AUC: 0.486). Additionally, we show that a number of predicted cancer-associated m^6^A sites are supported by existing literatures, suggesting that the proposed approach can effectively uncover the underlying epitranscriptome circuits of disease mechanisms. An online database DRUM, which stands for **d**isease-associated **r**ibon**u**cleic acid **m**ethylation, was built to support the query of disease-associated RNA m^6^A methylation sites, and is freely available at: www.xjtlu.edu.cn/biologicalsciences/drum.

## Introduction

Epigenetic regulation, such as, RNA methylation, DNA methylation and post-translational modification (PTM), participates in a variety of important cellular processes, including embryonic development, maintenance of chromosome stability and X-chromosome inactivation (Wu and Zhang, 2014). Over the past decade, DNA methylation has been considered to play a critical key role in gene expression regulation to moderate various biological functions. It has been found that dysregulated DNA methylation is associated with various diseases. For example, epigenetic defects, like the global genomic hypo-methylation or locus-specific hyper-methylation is one of the cancer hallmarks (Gopalakrishnan et al., 2008). To date, there have been a number of works seeking to unveil the functional relevance of epigenetic modifications to various diseases. DiseaseMeth (Xiong et al., 2016) contains aberrant DNA methylation in 679602 disease-gene association collected from 32701 samples; MethyCancer (He et al., 2007) and MethHC (Huang et al., 2014) supports the query of cancer and disease related DNA methylation profiles. ActiveDriverDB (Huang et al., 2014), CaspNeuroD (Kumar and Cieplak, 2016), dbPTM (Huang et al., 2019) and PTMSNP (Kim Y. et al., 2015) investigated human disease mutations that potentially functional through post-translational modifications. Recently, Xu and Wang investigated the disease-associated phosphorylation sites of protein from a multi-layer heterogeneous network using the random walk algorithm (Xu and Wang, 2016). These studies greatly advanced our understanding of the role epigenetic modifications play in disease pathology. However, the study of biochemical modifications have been dominated by DNA methylation and post-translation protein modifications, until recently, RNA methylation emerged as important layer for gene expression regulation.

Firstly identified more 40 years ago (Wei et al., 1976), more than 100 different types of RNA modifications have also been discovered in cell as epigenetic mark recognized by other regulators for modulating the genetic information (Cantara et al., 2011; Boccaletto et al., 2017), among which, *N*^6^-methyladenosine is the most abundant in mRNA (Fu et al., 2014; Meyer and Jaffrey, 2014). A series of studies reveal that, RNA methylation plays a crucial role in the regulation of circadian clock (Fustin et al., 2013), RNA stability (Wang et al., 2014), cell differentiation (Geula et al., 2015), translation efficiency (Wang et al., 2015), as well as DNA damage response (Xiang et al., 2017) and cortical neurogenesis (Yoon et al., 2017). It has been shown that RNA methylation may be central in disease pathology especially in various cancers, including breast cancer (Cai et al., 2018), myeloid leukemia (Barbieri et al., 2017; Kwok et al., 2017; Li Z. et al., 2017; Vu et al., 2017), liver cancer (Chen M. et al., 2017), carcinoma (Li et al., 2017a), glioma (Visvanathan et al., 2017; Zhang et al., 2017), etc. (Hsu et al., 2017; Stojković and Fujimori, 2017; Wang S. et al., 2017). Recent studies revealed the impacts of m6A modification on specific diseases. E.g., N6-methyladenosine (m6A) modification of mRNA plays a role in regulating the self-renewal and tumorigenesis of glioblastoma stem cell (GSC). Studies report the knockdown of RNA methyltransferase complex METTL3 or METTL14 can dramatically decrease abundance of m6A methylation and alter mRNA expression of genes (e.g., ADAM19, EPHA3, KLF4), thereby promoting human GSC growth (Cui et al., 2017). Meanwhile, the up-regulation of RNA m6A demethylase ALKBH5 can also induce the proliferation of GSCs (Zhang et al., 2017). It is found that FOXM1, the cell cycle regulator, is the downstream target of m6A modification through inhibition of ALKBH5 by shRNA. Importantly, the hypo-methylation of target mRNA promotes the binding of RNA binding protein HuR, resulting in increased FOXM1 expression and the development of glioma (Zhang et al., 2017). Additionally, the RNA m6A demethylase FTO is found to be an oncogene of the Acute Myeloid Leukemia (Li Z. et al., 2017). studies show that reduced m^6^A levels in some mRNA transcripts, such as ASB2 and RARA, can enhance leukemic oncogene-mediated cell transformation, leukemogenesis, and inhibit AML cell differentiation (Li Z. et al., 2017). Furthermore, Zhang et al. found that the breast cancer cells stimulated by hypoxia can cause upregulation of m6A demethylase ALKBH5 expression, which is mediated by hypoxic induction factor (HIF). Consequently, it results in the demethylation of the multipotent factor NANOG's mRNA, and hypomethylation increases the stability of mRNA so as to causes high expression of NANOG, further inducing the maintenance and metastasis of tumor stem cells (Zhang et al., 2016a).

Despite the growing interests in m^6^A RNA modification and its potential regulatory role in various diseases, the study of m^6^A methylation under the context of diseases has been restricted. The experimental approaches are mostly limited to the study of m^6^A mediator genes, i.e., the RNA methyltransferase (writer), demethylase (eraser) and RNA binding protein (reader). For instance, the RNA m^6^A demethylase FTO is also found to play an important role in neurogenesis, as well as in learning and memory. Hence, m^6^A modification is regarded to be related to Alzheimer's disease (Li L. et al., 2017). And, another study reports RNA m^6^A demethylase ALKBH5 can relate to the major depressive disorder in Chinese Han population (Du et al., 2015). These studies are often less detailed in genomic resolution and could not unveil the disease relevance of a specific RNA methylation site. Comparing with the research dedicated to the experimental investigation of m^6^A site regulatory functions, bioinformatics is a possible method to identify the putative disease association of the m^6^A sites, thereby urgently needed at present. Till this day, the computational approaches for studying the association between m^6^A methylation and diseases have been limited to the disease-associated mutations that may potentially disrupt or form an m^6^A-containing motif, which may be regulated through epitranscriptome layer. Works of this category include m6AVar (Zheng et al., 2018), which contains a number of functional variants involved in m^6^A modification, and m6ASNP (Jiang et al., 2018; Mo et al., 2018; Zhang et al., 2019), which is a tool for annotating genetic variants from the perspective of impact on m^6^A modification. Although generated fruitful results (Mo et al., 2018,a,b), SNP-based approaches are limited to existing GWAS analysis results and cannot predict previously unknown novel associations between m^6^A sites and diseases. Other disease association study of the epitranscriptome focuses on a specific mediator gene of the epitranscriptome, which could cover the disease association of the epitranscriptome for only a limited number of diseases (Zhang et al., 2016b, 2019), but not yet an arbitrary disease.

The accumulation of epitranscriptome high-throughput sequencing data has provided numerous possibilities for epitranscriptome analysis. Nowadays, the most widely used approach for profiling transcriptome-wide RNA methylation is methylated RNA immunoprecipitation sequencing (m^6^A-seq or MeRIP-seq) (Wan et al., 2015), and the technique has been used in various studies to profile the condition-specific RNA methylation (Liu H. et al., 2018; Xuan et al., 2018). The m^6^A RNA methylation sites has been more accurately identified in human, mouse and other species with the machine learning approaches. It is possible and solely needed to develop computational approaches for understanding the disease relevance of individual RNA methylation sites by taking advantage of the large amount of epitranscriptome data accumulated from existing studies (Chen X. et al., 2017; Chen et al., 2019). Random walk on a multi-layer network has been used previously to uncover the important role of RNA molecules under a pathologic context, including disease-related long non-coding RNAs (lncRNA) (Zhou et al., 2015) and miRNAs (Mendell and Olson, 2012). In the field of epitranscriptome analysis, random walk with start (RWR) algorithm has been implemented to study the functional protein-protein network driven by RNA methylation enzymes through the regulation of epitranscriptome layer (Zhang et al., 2016b).

In this work, we for the first time extracted disease-associated m^6^A sites through a multi-layer heterogeneous network using random walk with restart (RWR) algorithm, and provided with a more specific regulatory circuit that functions at epitranscriptome layer. Specifically, a novel multi-layer heterogeneous network was constructed from gene expression and RNA methylation data. The nodes of the network are corresponding to the diseases, the genes and the m^6^A RNA methylation sites. The network contains both cross-layer associations, such as gene-m^6^A site association, disease-gene association, as well as the with-layer associations, i.e., gene-gene association, m^6^A site-m^6^A site association and disease-disease association. Depending on the known gene-disease network and gene-m^6^A site network that link the m^6^A site and disease layers together, the potential relationships of the m^6^A sites and diseases are both implicated (Tong et al., 2008). The within-layer association networks (e.g., disease-disease association) can further enhance the confidence of interactions.

To evaluate the performance of the proposed approach, a 10-fold cross-validation was implemented. Our RWR-based predictor achieved a reliable prediction performance and the area under the receiver operating characteristic curve (AUC) is equal to 0.83, compared with an alternative hypergeometric test-based approach (AUC: 0.73) and a random predictor (AUC: 0.48). A website DRUM, which stands for **d**isease-related **r**ibo-n**u**cleic acid **m**ethylation, is built to support the query of the RNA methylation sites most probable related to 705 diseases. The DRUM website is freely available at: www.xjtlu.edu.cn/biologicalsciences/drum.

## Materials and Methods

**Figure 1:**
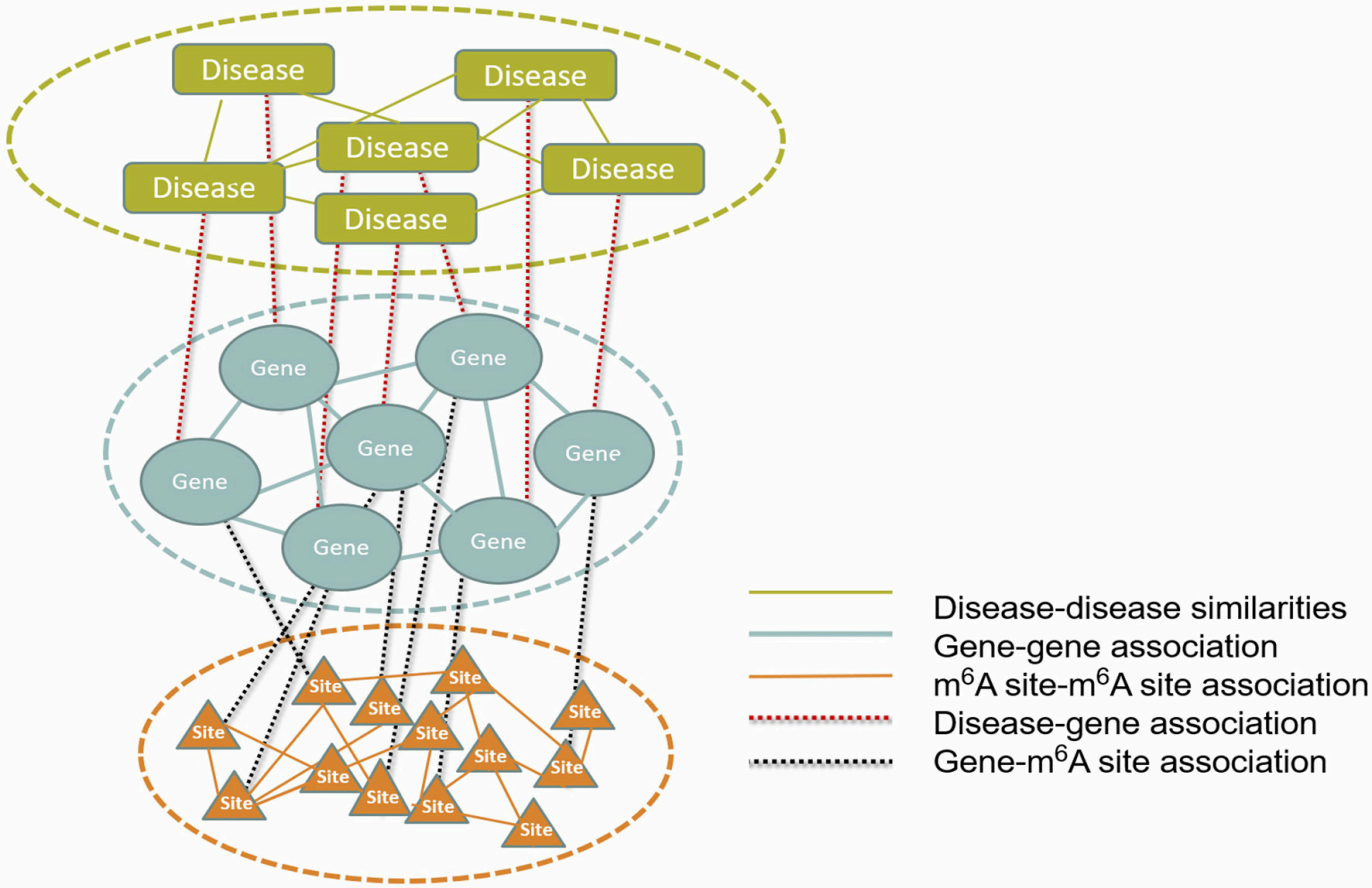
The constructed multi-layer heterogeneous network. To infer disease-m^6^A site association, a multi-layer heterogeneous network was constructed, which consists of three types of nodes, i.e., the disease, gene and m^6^A site, and five types of associations, i.e., gene-gene, gene-disease, gene-m^6^A site, disease-disease, and m^6^A site-m^6^A site.

To infer disease-associated RNA methylation site, a multi-layer heterogeneous network was constructed, which consists of three types of nodes, i.e., the diseases, genes and m^6^A sites, and five types of associations, i.e., gene-gene association, gene-disease association, gene-m^6^A site association, disease-disease association, and m^6^A site- m^6^A site association (see Figure 1). The network was constructed by integrating the RNA methylation profiles, the RNA expression profiles and gene-disease associations, which will be detailed in the next.

### RNA Methylation Data

The locus information of 477,452 m^6^A RNA methylation sites in human was extracted from RMBase V2 (Xuan et al., 2018), which collected the m^6^A RNA methylation sites reported by multiple techniques including m^6^A-seq, miCLIP, m^6^A-CLIP, and PA-m^6^A-seq (Li et al., 2017b). In the site filtering stage, 182,358 sites, which are supported by more than 10 experiments, are kept. To further select the most robust m^6^A methylation signal, we selected the methylation sites with average methylation level within the 70 percentile. Additionally, the m^6^A sites with the variance of methylation level ranked in the top 80 percentiles were retained, which represent the most actively regulated set of m^6^A sites, whose functional relevance may be more reliably inferred. In the end, 28278 RNA methylation sites were retained for further analysis.

Although there exists base-resolution m^6^A profiling techniques, technique either cannot be used for methylation level quantification (e.g., miCLIP and m^6^A-CLIP), or the limited number of available samples is insufficient to infer reliably the associations (e.g., PA-m^6^A-seq). Instead of using data generated from base-resolution techniques, the RNA methylation levels of each m^6^A sites were estimated from MeRIP-seq data, which profiled the m^6^A epitranscriptome under 38 different experimental conditions (see Table 1). The raw data was downloaded from GEO and aligned to human reference genome hg19 with HISAT2 (Kim D. et al., 2015). The reads associated with each RNA methylation sites were counted under R enrironment, and the methylation status were quantified using the M-value, which is essentially the log2 fold change of reads in the IP sample compared to the input control sample of MeRIP-seq data, as is shown in (1):

(1) $$\begin{array}{r} \text{M-value}=\log_{2}\left(\frac{\text{RPK}{\text{M}}_{IP}+0.1}{\text{RPK}{\text{M}}_{Input}+0.1}\right) \end{array}$$

where, RPKM_*IP*_ and RPKM_*Input*_ represent the reads abundance of a specific m^6^A site (101 bp flanked region) in the IP and Input control sample of MeRIP-seq data, respectively. The reads abundance was measured in terms of the Reads Per Kilobase of transcript per Million mapped reads (RPKM). When multiple biological replicates from the same experimental conditions were available, they were merged during the data processing stage. Quantile normalization was then performed to remove potential batch effect.

**Table 1:** MeRIP-seq data used in the analysis.

**Conditions**	**Cell type**	**Treatment**	**GEO number**	**References**
1–2	HEK293T	SYSY*; NEB*	GSE29714	Meyer et al., 2012
3–7	HepG2	Ultraviolet, heat shock, hepatocyte growth factor, interferon, control	GSE37005	Dominissini et al., 2012
8–9	U2OS	Control, 3-Deazaadenosine	GSE48037	Fustin et al., 2013
10–12	HeLa1	Control, METTL14 KO, WTAP KO	GSE46705	Wang et al., 2014
13–14	HeLa2	Control, METTL3 KO	GSE46705	Wang et al., 2014
15	hNPC		GSE54365	Schwartz et al., 2014
16	hESC		GSE54365	Schwartz et al., 2014
17–19	HEK293T	WTAP KD, METTL3 KD, control	GSE54365	Schwartz et al., 2014
20–22	OKMS	5 days after fully reprogrammed into iPSC induction with/without Dox, fully reprogrammed into iPSC	GSE54365	Schwartz et al., 2014
23–34	A549	WTAP KD, WTAP KD BR1, METTL14 KD, METTL14 KD BR1, METTL3 KD, METTL3 KD BR1, GFP KD, GFP KD BR1, KIAA1429 KD, METTL3 and METTL14 KD, control	GSE54365	Schwartz et al., 2014
35–36	H1A	Resting (undifferentiated) human H1-ESCs, 48 h of Activin A induction toward endoderm	GSE52600	Batista Pedro et al., 2014
37–38	H1B	Resting (undifferentiated) human H1-ESCs, 48 h of Activin A induction toward endoderm	GSE52600	Batista Pedro et al., 2014

*The MeRIP-seq data used in the analysis profiled the epitranscriptome under 38 different experimental conditions*.

* *SYSY and NEB are anti-m^6^A antibodies made by two different companies*.

### Gene Expression Data

The gene expression profiles under the same 38 experimental conditions, (matched with the RNA methylation data) were extracted from the input control samples of the MeRIP-seq data, which measures the expression level of genes. Similar to the processing of RNA methylation data, the gene expression levels were measured in RPKM, multiple biological replicates were merged, and the quantile normalization was performed to reduce batch effect.

### Disease-Gene Association

The human gene-disease associations used in our analysis were directly collected from OUGene, which collects the over- and under-expressed genes under a specific disease condition (Pan and Shen, 2016). A total of 41,269 associations between 705 human diseases and 1080 genes from OUGene were integrated into our multi-layer heterogeneous network.

### Disease-Disease Similarities

Since similar diseases are often associated with similar gene sets, the association between diseases was also considered (Xu and Wang, 2016). The disease-disease similarity network was constructed based on MeSH (medical subject headings vocabulary) terms (Lowe and Barnett, 1994), and the diseases share significant number of MeSH terms are considered more associated. Specifically, the similarity of two diseases *V*_*ij*_ is denoted by the number of shared MeSH terms panelized by the total number of terms in their disease titles, as shown in the following

(2) $$\begin{array}{r} V_{ij}=\frac{\left|d_{i}\cap d_{j}\right|}{\left|d_{i}\cup d_{j}\right|}, \end{array}$$

where, *d*_*i*_ and *d*_*j*_ strand for all the MeSH terms of the disease *i* and *j *, respectively. And |*| denotes the total number of terms. Please note that the OUGENE database does not contain the MeSH terms information. The MeSH terms associated with various diseases was extracted from the semantically integrated database of disease SIDD (Liang et al., 2013). No additional cut-off threshold was further applied. All the pair-wise associations between diseases were kept for the analysis.

### Association Between m^6^A Sites

The association between m^6^A RNA methylation sites was inferred from RNA methylation profiles. We speculate that the functions of two m^6^A sites are related if their methylation profiles are highly correlated across different experimental conditions. Fisher's asymptotic test was implemented to calculate the Pearson correlation coefficient (Pcc) *P*-Values for each m^6^A site pairs, and then Bonferroni multiple test correction was used for adjusting the *P*-Values. Only the m^6^A site pairs with the adjusted *P* < 0.05 cut-off and the homologous Pcc value ranked in the top or bottom 10 percentile were considered as associated in our network (Liao et al., 2011). Positive and negative correlations were not distinguished in the association network, which is because that the regulatory impact of m^6^A RNA methylation is complex. It may both enhances or decreases transcriptional expression level for different genes, making it difficult to distinguish the functional consequences of positive or negative correlation at epitranscriptome layer.

### Gene-Gene Association

We constructed the gene-gene association networks from RNA expression data. The genes that exhibit strong positive or negative correlation are considered functionally related in our multi-layer heterogeneous network. And it followed the same procedure of building the associations between m^6^A RNA methylation sites.

### Association Between m^6^A Sites and Genes

Similar to gene-association or m^6^A site-m^6^A site association, the association between m^6^A sites and genes was constructed from the correlation of their expression and methylation levels. If the methylation level of an m^6^A site and the expression level of a gene are highly correlated across different experimental conditions, we assume that the two are functionally related. The construction of gene-m^6^A site network follows the same procedure of m^6^A site-m^6^A site network.

### The Multi-Layered Heterogeneous Network

As shown in Figure 1, the multi-layer heterogeneous network incorporates three types of nodes and five types of associations, from which, it is possible to infer disease-associated m^6^A RNA methylation sites. We use *D*{*d*_1_, *d*_2_, ⋯ , *d*_*N*_},*S*{*s*_1_, *s*_2_, ⋯*s*_*M*_} and *G*{*g*_1_, *g*_2_, ⋯ , *g*_*T*_} successively to represent three types of nodes within network: the diseases, the m^6^A sites and the genes. And *N* , *M* and *T* denote the total number of diseases, m^6^A sites and genes, respectively. The associations within the disease, the gene and the site layer can then be represented by *DD*{*d*_*ij*_:*i, j* = 1, 2, ⋯ , *N*}, *GG*{*g*_*i, j*_:*i, j* = 1, 2, ⋯ , *T*}and *SS*{*s*_*ij*_:*i, j* = 1, 2, ⋯ , *M*}, respectively. While the other two types of connection between different types of nodes are represented by *DG*{*dg*:*i* = 1, 2, ⋯ , *N*; *j* = 1, 2, ⋯ , *T*} and *SG* {s*g*_*ij*_:*i* = 1, 2, ⋯ , *M*; *j* = 1, 2, ⋯ , *T*}. Please note that the missing information of m^6^A site-disease association is substituted by *DS* { *ds*_*ij*_:*i* = 1, 2, ⋯ , *M*; *j* = 1, 2, ⋯ , *N*}, which is a null network and used to complement the integrity of the adjacency matrix of the multi-layer heterogeneous network.

### Construct the Adjacency Matrix of the Overall Network

In RWR algorithm, the multi-layer heterogeneous network is represented by the *W* matrix. It is a column-normalized adjacency matrix and comprises of nine sub matrixes, which respectively reflects diverse relationships among the nodes (i.e., disease, gene, and m^6^A site). Among them, *M*_*DS*_, *M*_*SG*_,and *M*_*DG*_ strands for the probabilities of nodes transmitting between different type of nodes, and their transpose matrixes are denoted by *M*_*SD*_,*M*_*GS*_, and *M*_*GD*_, respectively. While *M*_*DD*_, *M*_*SS*_ and *M*_*GG*_ represent the transition probabilities among the same type of nodes. *M*_*GS*_,*M*_*GD*_,*M*_*DD*_, *M*_*SS*_, and *M*_*GG*_ were estimated previously; while *M*_*SD*_ is set to be **0**, as it is unknown. Due to the different weights used in various types of networks, the adjacency matrix were further normalized with

(3) $$W=\left[\begin{array}{ccc} \frac{1}{2}\times M_{DD}\; & \frac{1}{3}\times M_{GD}\; & 0\\ \frac{1}{2}\times M_{DG}\; & \frac{1}{3}\times M_{GG}\; & \frac{1}{2}\times M_{SG}\\ 0\; & \frac{1}{3}\times M_{GS}\; & \frac{1}{2}\times M_{SS} \end{array}\right]$$

where, all the 5 sub networks were assigned with the equal weight, despite that their relative importance may be further optimized (Xu and Wang, 2016).

### Random Walk With Restart (RWR) Algorithm

Random walk with start (RWR) algorithm, as an iterative network propagation method, was used for inference of disease-associated RNA methylation site on our multi-layer heterogeneous network. RWR algorithm is defined that a random walker starts from a specific node and iteratively transmits to its neighbor nodes. The pump flow of random workers is proportional to the weights of edge, and it is synchronously recycled to the initial position with the certain proportion. Compared to the conventional random walk approach, RWR algorithm allows the return of the random walkers, so that it can avoid all random walkers assembling at a single node location. When applied to multi-layer heterogeneous networks, another notable strength of RWR is that it does not restrict movement of the random walker among nodes of the same type, and allows walking among all the three layers of the network via the five types of edges. In the end, when the terminated condition is satisfied, all the reachable positions can obtain a steady-state probability, and the nodes are ranked according to the proportion that random walker reaches. Here, we assume the *P*_*s*_ is the stopping probability of random walker at each position after the *s*-th iteration, which can be calculated as following:

(4) $$\begin{array}{r} P_{S\text{+}1}=\left(1-r\right)\times W\times P_{S}+r\times P_{0} \end{array}$$

(5) $$\begin{array}{r} P_{S\text{+}1}-P_{S}\leq 10^{-10} \end{array}$$

where, *r* is the restart probability, indicating the proportion of random walkers being recycled at step, and is set to 0.75 arbitrarily. And *P*_0_ refers to the initial probability vector of seed node and *W* is a matrix that consists of transition probabilities of movement through different types nodes (discussed in the next). Here, the stopping criterion for iteration is the difference of probabilities between the (*S*+1)-th iteration and its prior iteration falls below a predefined threshold 10^−10^. We can have the disease node *d*_*i*_ as the seed node with initial probability 1, while the remaining disease nodes are assigned with an initial probability of 0. With the implementation of RWR algorithm, we can rank the disease-associated m^6^A sites according to the stable probability that the random walker *d*_*i*_ reaches each m^6^A site node.

**Figure 2:**
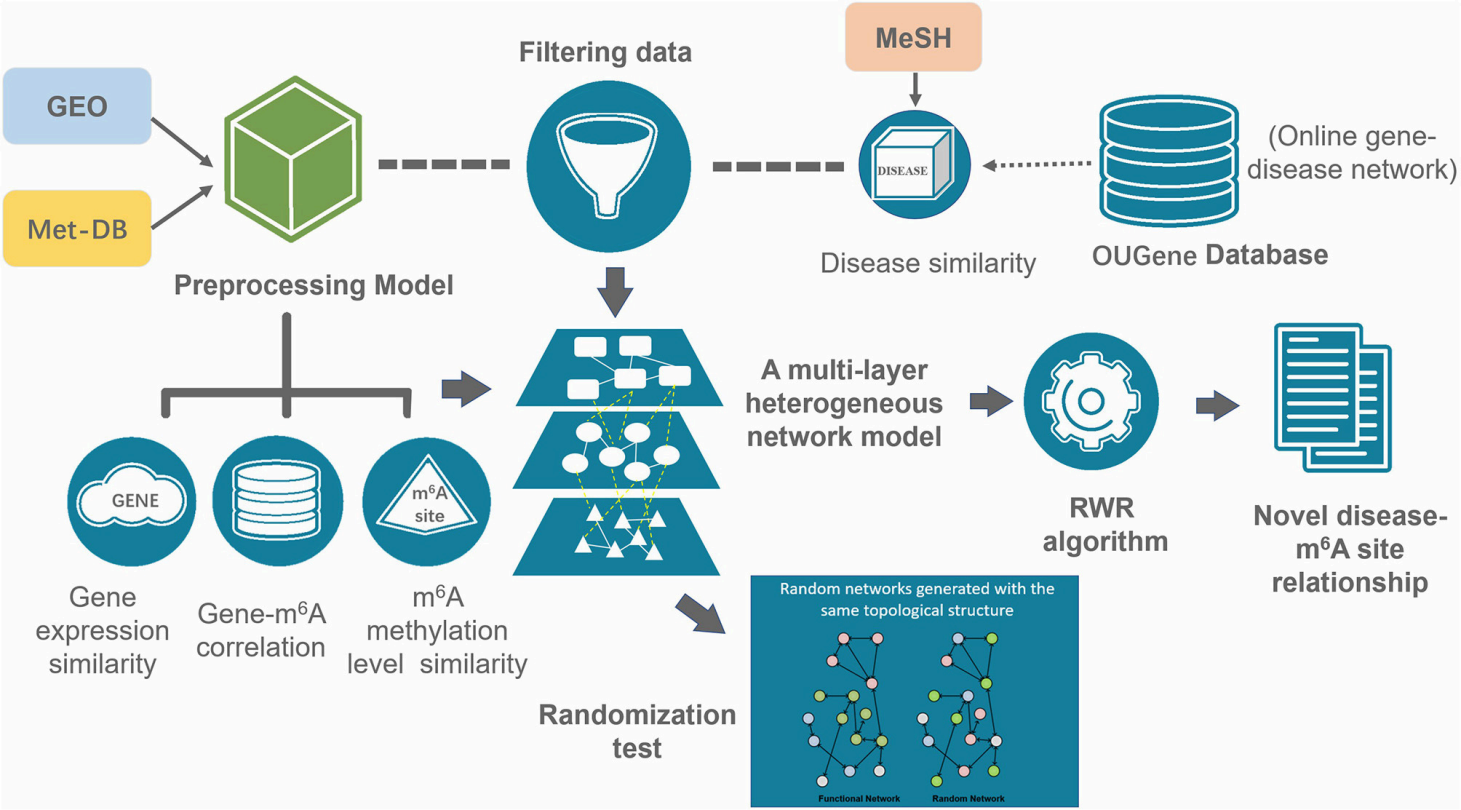
Overall workflow of the prediction. A multi-layer consists of three types of nodes (disease, gene and m^6^A site) was constructed from gene expression data, RNA methylation data, disease-gene association data and disease similarities. The disease-associated m6A RNA methylation sites were inferred with the RWR algorithm.

The overall RWR algorithm is summarized in the following (Figure 2).

### Evaluate the Statistical Significance of Prediction by Random Permutation

In general, of interests are the nodes with highest probabilities in RWR result, as they are regarded as highly accessible from the initial node, and thus denotes the association. To evaluate the statistical significance of the prediction results, a randomization-based estimation (Jia and Zhao, 2014) is implemented. Specifically, we generated 100 random networks by building random edges within the multi-layer heterogeneous network but still maintaining its original topology characteristics (Liao et al., 2011). This randomization chose two arbitrary edges (e.g., a-b and c-d) and exchanged them (e.g., with a-d and c-b), if the new links generated not already exist in the network after the node exchange. Then, for each of 100 random networks, RWR algorithm is applied and ranks all the m^6^A sites according to the probabilities of association to the disease. These probabilities represent the observed probabilities of a negative association between a disease and an m^6^A site, with which the statistical significance of a prediction from the real network can be assessed (Jia and Zhao, 2014).

## Determine the Direction of the Predicted Association

Given an m^6^A site is predicted to be associated with a disease, we would like to know whether we should expect a hyper or hypo-methylation of this site under disease condition. Conceivably, if the methylation level of this site is positively correlated to the genes that are overexpressed under disease condition, or anti-correlated to genes that are under expressed under disease condition, the site is likely to be hyper-methylated under disease condition; and vice versa. The median of the correlations of this site to all the disease-associated genes was used to infer the direction of the association, and has been provided at our website.

### An Alternative Approach for Performance Comparison

**Figure 3:**
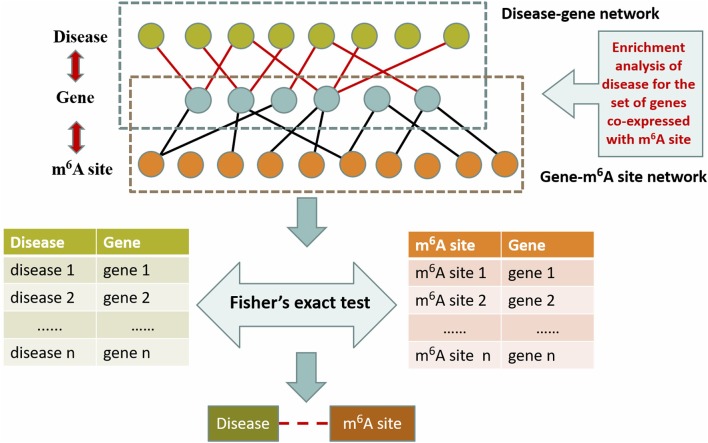
Hypergeometric test-based approach. This method is based on the disease-gene association from OUGene database and the gene-m^6^A site association networks derived from the gene expression profiles and RNA methylation profiles. The statistical significance is assessed with the hypergeometric test.

To evaluate the performance of this approach, we also considered a naïve hypergeometric test-based approach, which assesses the association between a disease and an m^6^A sites by checking whether they are simultaneously linked to a significant number of genes in the constructed multi-layer heterogeneous network (see Figure 3). The statistical significance (P-Value) of the association can be assessed with a hypergeometric test, with

(6) $$\begin{array}{r} p\left(Y\geq y\right)=1-\overset{y-1}{\underset{i=0}{\sum }}\frac{C_{m-x}^{n-i}}{C_{m}^{n}} \end{array}$$

where, *m* denotes the total number of genes in the analysis. *n* denotes the number of genes linked to a specific disease in the gene-disease association sub network, *x* denotes the number of genes linked to a specific m^6^A site in the gene-m^6^A site sub network; and *y* denotes the number of genes associated with both the disease and the m^6^A site. With the *P*-Values, it is then possible to predict the disease-associated RNA methylation sites given a specific significance level. Please note that the above alternative approach takes advantage of only two out of the five types of associations: the gene-m^6^A site associations and disease-gene associations.

## Result

### Constructed Multi-Layer Heterogeneous Network

Utilizing the aforementioned approaches, a multi-layer heterogeneous network was constructed to incorporate three types of nodes (m^6^A site, gene, and disease) and five types of associations. The numbers of nodes and edges in each layer of the network were summarized in Table 2.

**Table 2:** Multi-layer heterogeneous network.

**Network**	**Nodes**	**Edges**
Disease-disease association	705	111735
Disease-gene association	1785	5246
Gene-gene association	1080	237772
Gene-m^6^A site association	29358	7161
m^6^A site- m^6^A site association	2827	64014

### Performance Evaluation

We employed the 10-fold cross-validation to evaluate the performance of the proposed RWR algorithm. During each iteration, 10% of disease-gene associations were deleted from the original multi-layer heterogeneous network and reserved as the testing data, while the remaining 90% of associations were used as training dataset.

The proposed approach was also compared to a random predictor, which is constructed by random permutation of the multi-layer heterogeneous network, and an alternative hypergeometric test-based approach.

To compare the performances of the different methods, the receiver operating characteristics (ROC) curve was implemented to illustrate the true positive rate (TPR) vs. the false positive rate (FPR) at different stringency cut-offs, and the performance of different methods can be measured by the area under the ROC curve (AUC).

**Figure 4:**
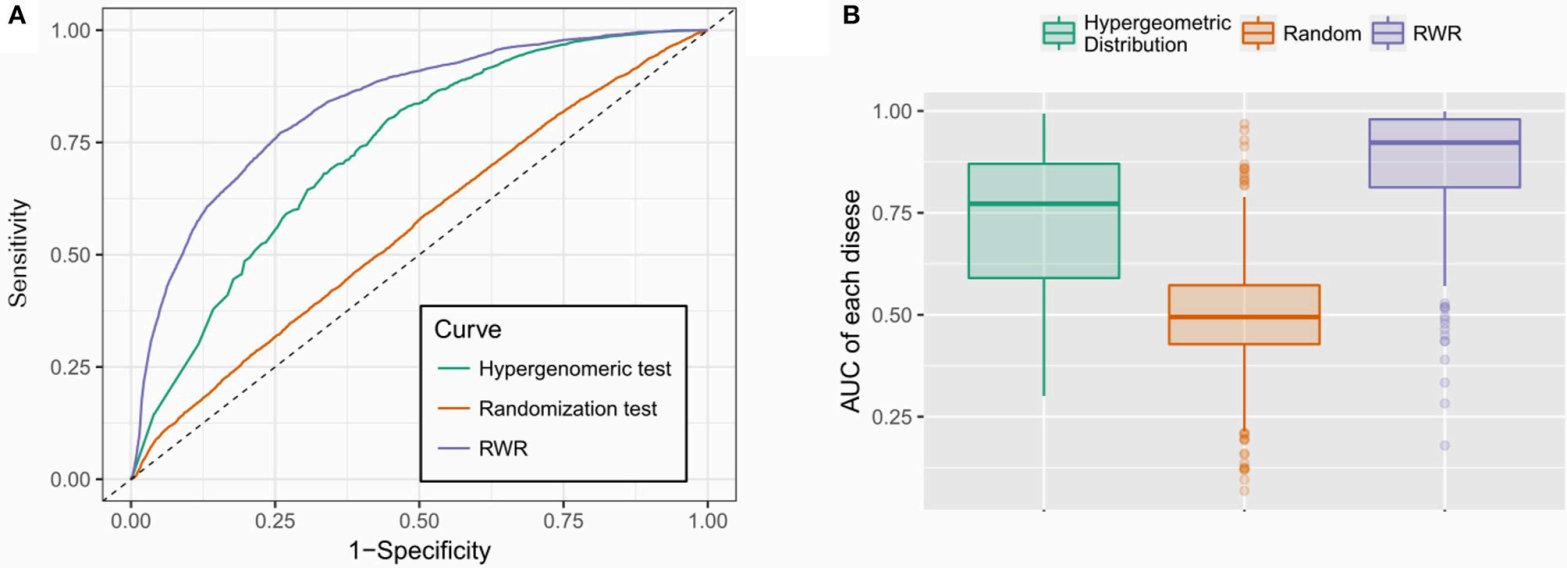
Performance evaluation. **(A)** RWR method achieved an AUC of 0.827, outperformed the hypergeometric test-based approach (AUC: 0.733) and the random predictor (AUC: 0.550); **(B)** RWR algorithm achieved superior performance on most of the diseases (average and median AUC: 0.867 and 0.913), compared to the other two methods: Hypergeometric test-based approach (average and median AUC: 0.723 and 0.772) and random predictor (average and median AUC: 0.486 and 0.479).

As is shown in Figure 4, the RWR method achieved an AUC of 0.827, outperformed the hypergeometric test-based approach (AUC: 0.733) and the random predictor (AUC: 0.550), which is close to the theoretical random performance (Figure 4A). Additionally, we also calculated the AUCs of each individual disease. As is shown in Figure 4B, RWR algorithm achieved superior performance on most of the diseases (average/median AUC: 0.867/0.913), compared to the other two methods: Hypergeometric test-based approach (average/median AUC: 0.723/0.772) and random predictor (average/median AUC: 0.486/0.479). This suggested that the multi-layer network model coupled with RWR algorithm could effectively predict the disease-m6A site associations, or potentially unveil the disease circuits regulated at epitranscriptome layer.

The prediction results are relatively reliable on the following diseases (Table 3), and they may be more relevant to epitranscriptome regulation.

**Table 3:** Diseases achieved highest accuracy.

**Disease**	**AUC**	**# of Sites**
Prostate cancer	0.808	130
Hepatocellular carcinoma	0.842	121
Glioblastoma	0.801	68
Hypertension	0.847	44
Alzheimer's disease	0.828	41
Osteosarcoma	0.840	40

### Case of Study: Cancer-Related m^6^A Sites

**Figure 5:**
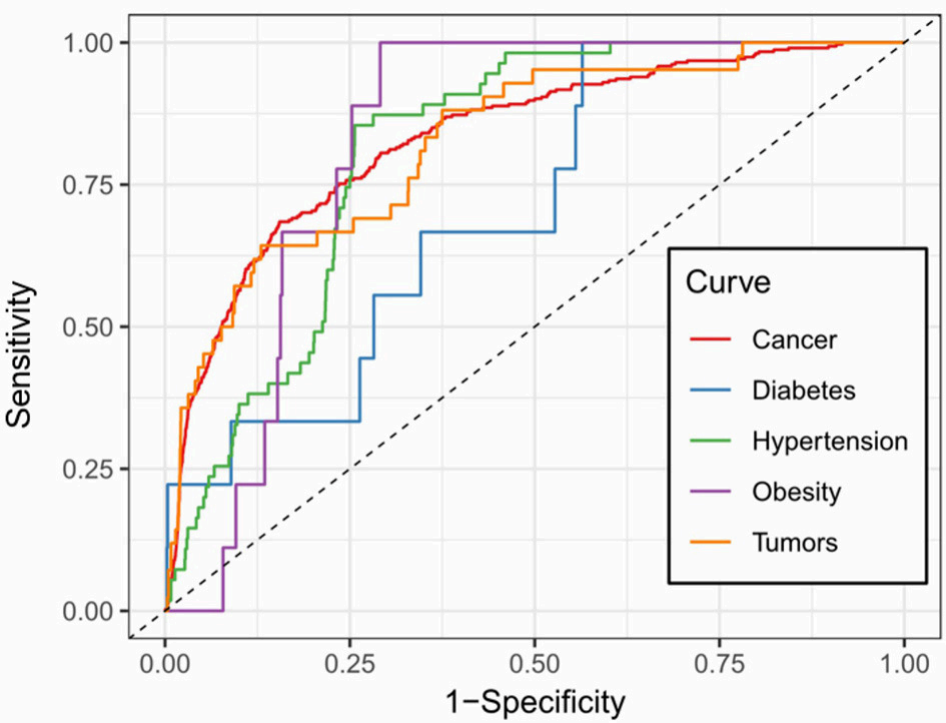
Prediction accuracy of five common m^6^A site-associated diseases. Figure shows the accuracy of disease associated m^6^A sites for five common diseases, including cancer (AUC: 0.832), diabetes (AUC: 0.717), hypertension (AUC: 0.812), obesity (AUC: 0.828) and tumors (AUC: 0.825), respectively. Among them, the prediction of cancer-related m^6^A sites achieved relatively stable performance.

We further examined the prediction performance of several common diseases. For top 100 predictions, the proposed approach achieved reasonable performance in all the 5 diseases tested (Table 4). As is shown in Figure 5, the cancer-related m^6^A site prediction achieved relatively steady performance. Indeed, recent studies suggest that m^6^A RNA methylation plays a crucial role in the pathologies of breast cancer, myeloid leukemia, liver cancer, carcinoma, glioma, etc. (Hsu et al., 2017; Stojković and Fujimori, 2017; Wang S. et al., 2017). Additionally, the model works better on cancer may partially due to the samples used are mostly related to cancer and tumor (see Table 1). As cancer samples were used, cancer-specific functions are more easily inferred from the data available. However, the samples were collected unbiasedly from all the published studies. The collection only reflects that most existing m^6^A-seq studies are either based on cancer cell lines or related to cancer. It suggests that inferring cancer-associated m^6^A sites may be more feasible than other diseases with the data cumulated from existing studies. We thus used cancer-related m^6^A sites in the next for a case study by checking whether our predictions are supported by existing literatures. Interestingly, many of our predicted associations are supported (see Table 5).

**Table 4:** Number of hits for top 50 predictions of a disease.

	**Tumors**	**Cancer**	**Obesity**	**Diabetes**	**Hypertension**
Hits in prediction	8	65	1	2	6
By Random	0.49	3.64	0.10	0.10	0.64
Total	42	314	9	9	55
Enrichment	16.44	17.87	9.59	19.18	9.42
*p*-value*	8.495E-4	1.394E-20	0.225	8.361E-3	0.371

*The p-values are calculated from binomial test*.

**Table 5:** Cancer-associated m^6^A sites supported by literature.

**Site ID**	**Host Gene**	**References**
m6A_site_102214	GLI1	Das et al., 2009; Carpenter and Lo, 2012
m6A_site_103132	SRGAP1	Feng et al., 2016
m6A_site_98274	BCDIN3D	Yao et al., 2016
m6A_site_96139	PRICKLE1	Chan et al., 2006; Daulat et al., 2016
m6A_site_90049	WNK1	Shyamasundar et al., 2016
m6A_site_81948	GDPD5	Wijnen et al., 2014; Cao et al., 2016
m6A_site_82683	KCTD21	Li et al., 2016
m6A_site_84205	KDM4D	Berry and Janknecht, 2013; Soini et al., 2015
m6A_site_85170	ALKBH8	Shimada et al., 2009; Ohshio et al., 2016
m6A_site_85220	CUL5	Fay et al., 2003; Burnatowska-Hledin et al., 2004
m6A_site_85837	DIXDC1	Wang et al., 2009; Cong et al., 2016
m6A_site_81777	XRRA1	Mesak et al., 2003; Wang W. et al., 2017
m6A_site_49878	ZEB1	Spaderna et al., 2008; Schmalhofer et al., 2009

Additionally, there are cases when dysregulated RNA methylation status is observed but does not lead to RNA level differential expression. Such associations may still be predicted by the proposed approach. DRUM works directly with RNA methylation data, and can thus detect associations that are observable at epitranscriptome layer only (see Table 6).

**Table 6:** Epitranscriptome layer association with diseases.

**Disease**	**Host gene of m^6^A site**	**References**
Non-small cell lung carcinoma	CENPE, BTN3A1, LMBR1 and KBTBD11	Lin et al., 2016
Leukemia	BCL2, CYP1A1, CD83 and ZNF445	Bansal et al., 2014; Chen X. et al., 2017; Li Z. et al., 2017; Vu et al., 2017
Endometrial cancer	PHLPP2	Liu J. A. et al., 2018
Glioma	POU3F2	Visvanathan et al., 2017

*The RNA transcripts of these genes are differentially methylated under the disease condition, but are not differentially expressed (at transcriptional level) according to the OUGene database (Pan and Shen, 2016). Their associations to tumors and cancers were correctly predicted by the proposed approach*.

To gain more insights, the m^6^A-seq data from Non-Small Cell Lung Cancer (NSCLC) cell line (A549) and the normal control cell line (H1299) were obtained (Lin et al., 2016). Differential RNA methylation analysis and differential expression analysis were performed using exomePeak R/Bioconductor package and the Cuffdiff software, respectively, with their default settings. The results are then compared to the predictions from the proposed approach. In the end, 9 sites predicted to be associated with NSCLC were validated (Please see Supplementary Materials for more details), including one site located on the gene ING5) that shows no differential expression (log2 fold change = 0.07, FDR = 0.999) but significant differential methylation (log2 fold change = 0.762 and FDR = 0.027) (see Figure S3).

Altogether, our case studies indicate that the proposed method is effective in uncovering putative disease-m^6^A site associations, especially cancer-related m^6^A sites. The approach we developed may be useful to unveil the molecular pathologies regulated at epitranscriptome layer and provide potentially new perspective for effective therapeutic strategies of cancer and other diseases.

### DRUM: Database for Disease-Associated RNA Methylation

**Figure 6:**
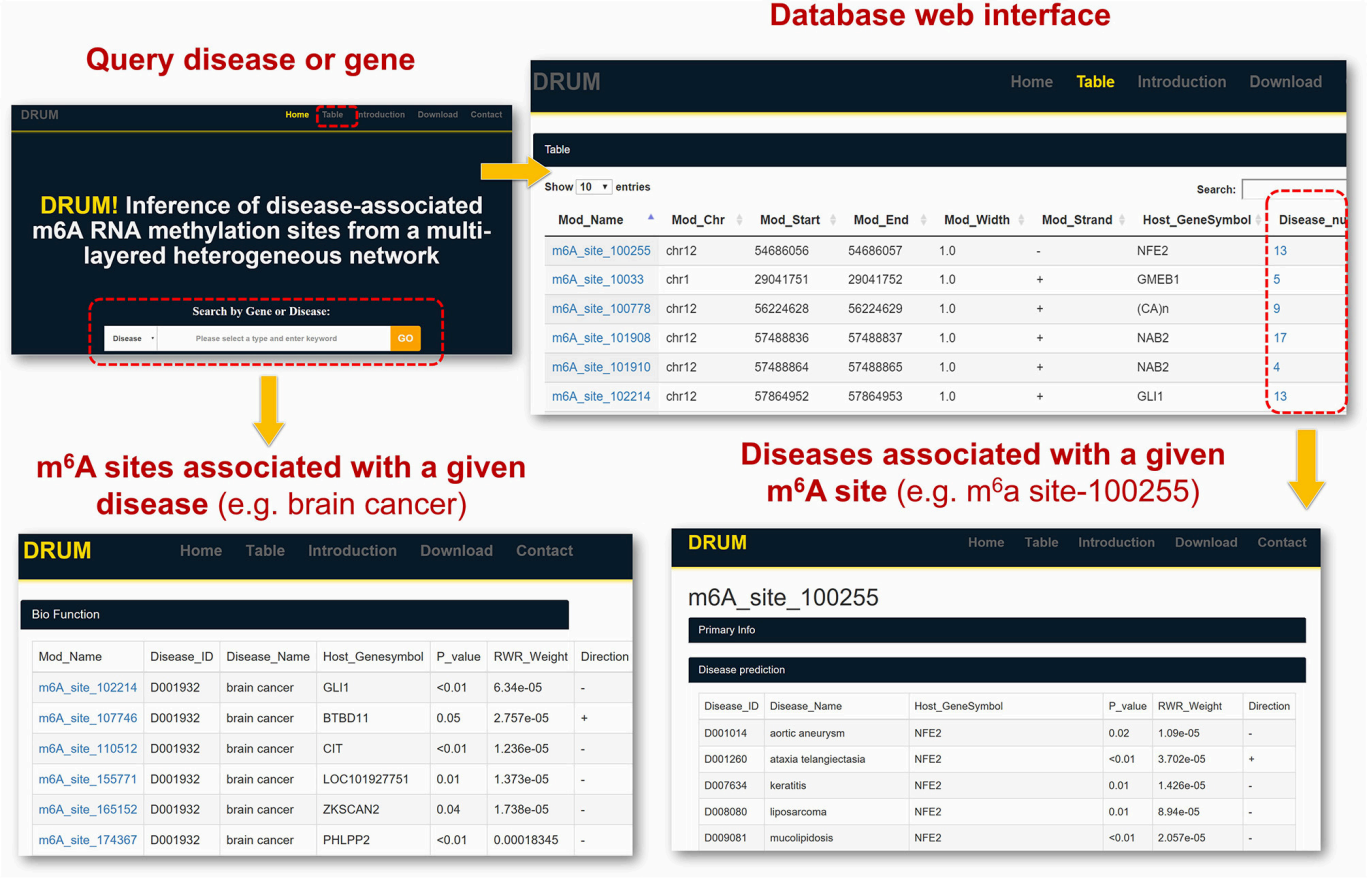
DRUM Database. DRUM is a public online database for disease-associated m^6^A sites. It integrates the m^6^A sites predicted to be associated with 705 diseases. The statistical significance of the prediction was assessed by random permutation. Users can access the data via the name of disease or the hosting gene of m^6^A site. It also supports the download of the entire prediction results for automated large-scale analysis.

To facilitate the exploration and direct query of our predicted results by the research community of RNA epigenetics, we developed an online database DRUM, which stands for **d**isease-associated **r**ibon**u**cleic acid **m**ethylation. The website hosts the top 100 m6A sites predicted to be associated to 705 diseases at significance level of 0.1, and supports queries that may be a disease or the host gene of m6A site (see Figure 6). Additionally, the prediction results can be downloaded in batch for large-scale automated analysis such as result comparison. The DRUM website is freely available at: www.xjtlu.edu.cn/biologicalsciences/drum.

## Conclusions

Investigation of *N*^6^-methyladenosine (m^6^A) RNA modification over the past 4 decade, especially since 2012, has uncovered its critical biological functions in various cellular processes. It has been clearly shown that RNA modifications directly or indirectly contribute to disease development and play a critical role in the many diseases such as cancers (Deng et al., 2017; Wang S. et al., 2017) and virus infections (Gokhale and Horner, 2017). It is solely needed to cover the epitranscriptome perspective of disease pathology or unveil the regulatory circuit of diseases regulated from epitranscriptome layer.

We presented here a multi-layer heterogeneous network model coupled with the RWR algorithm, which effectively incorporated five types of association among the diseases, genes and m^6^A sites, to unveil the disease association of individual m^6^A RNA methylation sites. To evaluate the performance of the proposed approach, a ten-fold cross-validation was performed. Superior performance is achieved by our approach (overall AUC: 0.827, average AUC 0.867) compared with the hypergeometric test-based approach (overall AUC: 0.7333 and average AUC: 0.723) and the random predictor (overall AUC: 0.550 and average AUC: 0.486). And a number of cancer-related RNA methylation sites are validated from existing studies. At last, an online database DRUM was constructed to enable the query of top m^6^A sites related to 705 different diseases.

**Figure 7:**
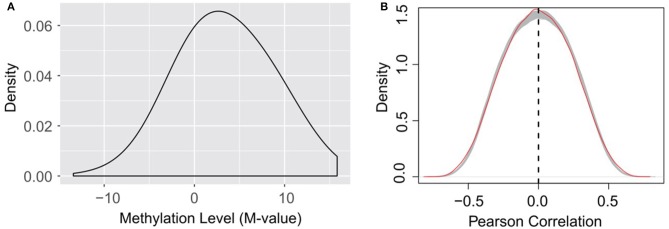
**(A)** Distribution of RNA methylation level (M-value). The estimated methylation levels are not strictly centered around 0, suggesting that the formation of M-value, which penalize lowly expressed transcripts as suggested by equation (1), may induce bias to the estimated methylation profiles on very lowly expressed transcripts. **(B)** Little linear correlation is observed between gene expression and RNA methylation profiles. The red line indicates the self-gene Pearson correlation coefficients, which are the correlation between the methylation level of a site and the expression level of its hosting gene. The gray lines indicate the Pearson correlation between the methylation level of a site and the expression level of a random gene under the 38 experimental conditions, when the methylation data and expression data are strictly separated, and thus independent from each other. A total of 1,000 gray lines were obtained from 1,000 random permutations, and serve as a null model of Pearson correlation distribution. The methylation level of an m^6^A site is not more linearly correlated (or anti-correlated) to the expression level of its host gene than a random gene.

It is worth noting that, as indicated in equation (1), the calculation of RNA methylation profiles partially relies on the expression data, which inevitably induces dependency between them. Ideally, we want to use independent datasets that profile RNA methylation and expression, respectively. Additionally, the detectability of methylated molecule depends on the abundance of transcripts, i.e., if the expression level of a specific transcript is low, it is not possible to accurately determine the methylation level (M-value) of the m^6^A sites on it. The current formulation of methylation level, as shown in equation (1), will penalize those very lowly expressed transcripts, and reports an *M*-value close to 0, which may induces additional bias to methylation profiles (as shown in Figure 7A).

Nevertheless, dispute of the bias and dependency that may be induced to the data, we didn't observe linear correlation (or anti-correlated) between the expression of the methylation level of an m^6^A site and the expression level of its host gene. The methylation level of an m^6^A site is not more linearly correlated (or anti-correlated) to the expression level of its host gene than a random gene (see Figure 7B). As suggested by a previous study, the epitranscriptome regulation changes only the percentage of methylated molecule, while transcriptional regulation changes only the abundance (Meng et al., 2014). Although slightly affecting each other, the two regulation mechanisms are observed to be largely independent and simultaneously regulate the transcriptome and epitranscriptome, which is consistent with our observation. As little linear correlation is observed between RNA methylation and gene expression profiles, and the association network was built based on Pearson correlation that relies on linear correlation (see section **Materials and Methods**), the predicted patterns associated with m^6^A sites are not likely to be dominated by gene expression profiles.

**Figure 8:**
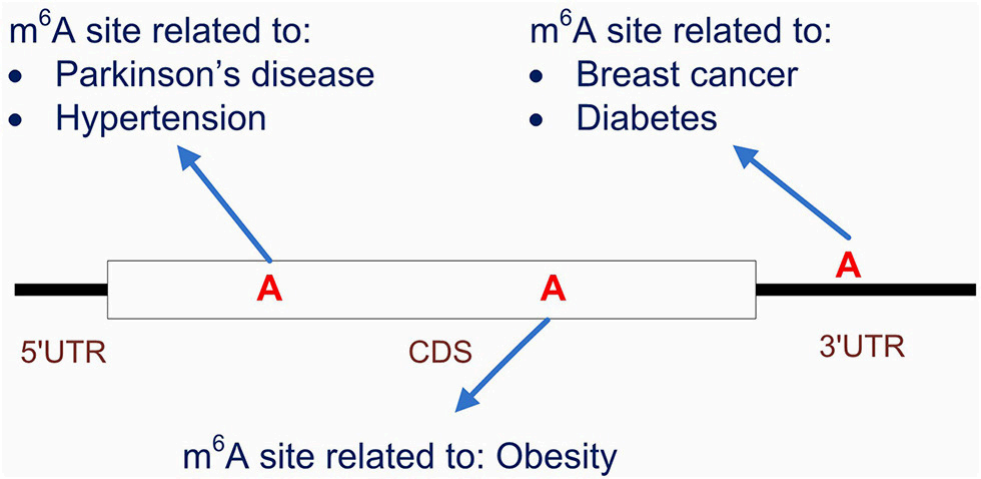
Predicting the disease-associated m^6^A sites. Our computational framework aims to predict disease-associated m^6^A sites. It is possible that multiple sites located on the same transcripts are associated to different diseases. Compared to general disease-gene association prediction, the proposed framework provides a more specific circuit of disease mechanism that functions at epitranscriptome layer.

It is also worth noting that, by starting from the methylation profiles of individual m^6^A sites, our work focused specifically on the disease circuits that are potentially regulated at epitranscriptome layer at the resolution of individual m^6^A sites (see Figure 8). This work is substantially different from general disease-gene association prediction, where the gene and disease may interact at any layer of gene expression regulation, such as at transcriptional or post- transcriptional layer (Chen and Yan, 2013). The work is also quite different from existing works (Zhang et al., 2016b, 2019) that aimed to predict diseases that may be significantly regulated at epitranscriptome layer, because these studies unveiled only the potential association between diseases and m^6^A RNA methylation, but didn't answer specifically which m^6^A sites are involved in the regulation process. Compared to existing works, our computational framework provided a more specific resolution for the study of disease mechanism functions at the epitranscriptome layer.

This presented computational scheme can be easily extended in the future by incorporating additional data sources such as disease-related functional variants involved in m^6^A modification (Jiang et al., 2018; Zheng et al., 2018), the regulatory specificity of the RNA methyltransferases and demethylases (Liu H. et al., 2018), or the associations between m^6^A site to other biomolecules (Xuan et al., 2018), so as to further improve prediction accuracy. Additionally, the method can be conveniently applied to other RNA modification types such as m^1^A (Dominissini et al., 2016) and Pseudouridine (Cabili et al., 2011) as well in other species such as mouse and yeast when sufficient amount of data is available.

## Data Availability

Publicly available datasets were analyzed in this study. This data can be found here: http://www.csbio.sjtu.edu.cn/bioinf/OUGene/.

## Author Contributions

JM and YT conceived the idea and initialized the project. ZW, YT, and BS processed the raw data. YT and XW constructed the network. YT implemented prediction, the performance evaluation and the case studies. KC built the website. YT drafted the manuscript. All authors read, critically revised, and approved the final manuscript.

### Conflict of Interest Statement

The authors declare that the research was conducted in the absence of any commercial or financial relationships that could be construed as a potential conflict of interest.

## Abbreviations

m^6^A: *N*^6^-methyladenosine
MeRIP-Seq: methylated RNA immunoprecipitation sequencing
IP: immunoprecipitation
DRUM: disease-associated ribonucleic acid methylation
ROC: receiver operating characteristics
AUC: area under the ROC curve
Pcc: Pearson correlation coefficient.
