# Supplementary material for: DRUM: Inference of Disease-Associated m6A RNA Methylation Sites From a Multi-Layer Heterogeneous Network
Source: Front Genet. 2019 Apr 3;10:266. doi: 10.3389/fgene.2019.00266 (PMC6456716; doi:10.3389/fgene.2019.00266)
Supplement: Supplementary file 1 [file Data_Sheet_1.PDF]

Supplement Materials

Table S1. Validated disease-associated RNA methylation sites under Non-Small Cell Lung Cancer

Gene	Chr	Start	End	Strand	Log_{10} FDR	Log_2 Fold Change	Significant
**ANKS1A**	chr6	34985661	34985662	+	-3.44	1.43	Yes
**HCFC1**	chrX	153236697	153236698	-	-10.8	0.877	Yes
**ING5**	chr2	242664525	242664526	+	-1.57	0.762	Yes
**MAML3**	chr4	141075022	141075023	-	-2	3.81	Yes
**PEX13**	chr2	61245649	61245650	+	-2.32	1.52	Yes
**PKD1**	chr16	2139686	2139687	-	-27	4.27	Yes
**PPIL2**	chr22	22051797	22051798	+	-14.2	0.913	Yes
**SMAD6**	chr15	66995541	66995542	+	-30.3	4.12	Yes
**SPSB1**	chr1	9428073	9428074	+	-54.4	2.43	Yes

Note: These 9 sites were reported to be differentially methylated under Non-Small Cell Lung Cancer after multiple hypothesis correction, i.e., FDR < 0.05. Differential RNA methylation analysis of Non-Small Cell Lung Cancer (A549 cell line) was performed again normal control (H1299 cell line) with exomePeak under the default settings. The original raw data was downloaded directly from GEO and aligned with Tophat2 under its default setting.

Table S2. The gene profiles of the genes that are differentially methylated under NSCLC

Gene	Control	NSCLC	Log_2 Fold Change	P_Value	FDR	Significant
**ANKS1A**	6.85529	3.94409	-0.79753	0.5228	0.99932	No
**HCFC1**	61.6017	37.2002	-0.72766	0.50725	0.99932	No
**ING5**	5.7628	6.06391	0.073478	0.9609	0.99932	No
**MAML3**	1.30381	6.06636	2.2181	0.10905	0.99932	No
**PEX13**	3.57623	7.88301	1.14031	0.5258	0.99932	No
**PKD1**	2.60854	7.44019	1.5121	0.3719	0.99932	No
**PPIL2**	25.615	14.8019	-0.79121	0.5479	0.99932	No
**SMAD6**	1.24646	47.2368	5.244	0.00615	0.152976	No
**SPSB1**	4.09593	15.1661	1.88859	0.13305	0.99932	No

Note: The data shows the expression level of the hosting genes for the RNA methylation sites in Table S1. The differential expression was performed between Non-Small Cell Lung Cancer (A549 cell line) and the normal control (H1299 cell line) with Cuffdiff2 under its default setting.

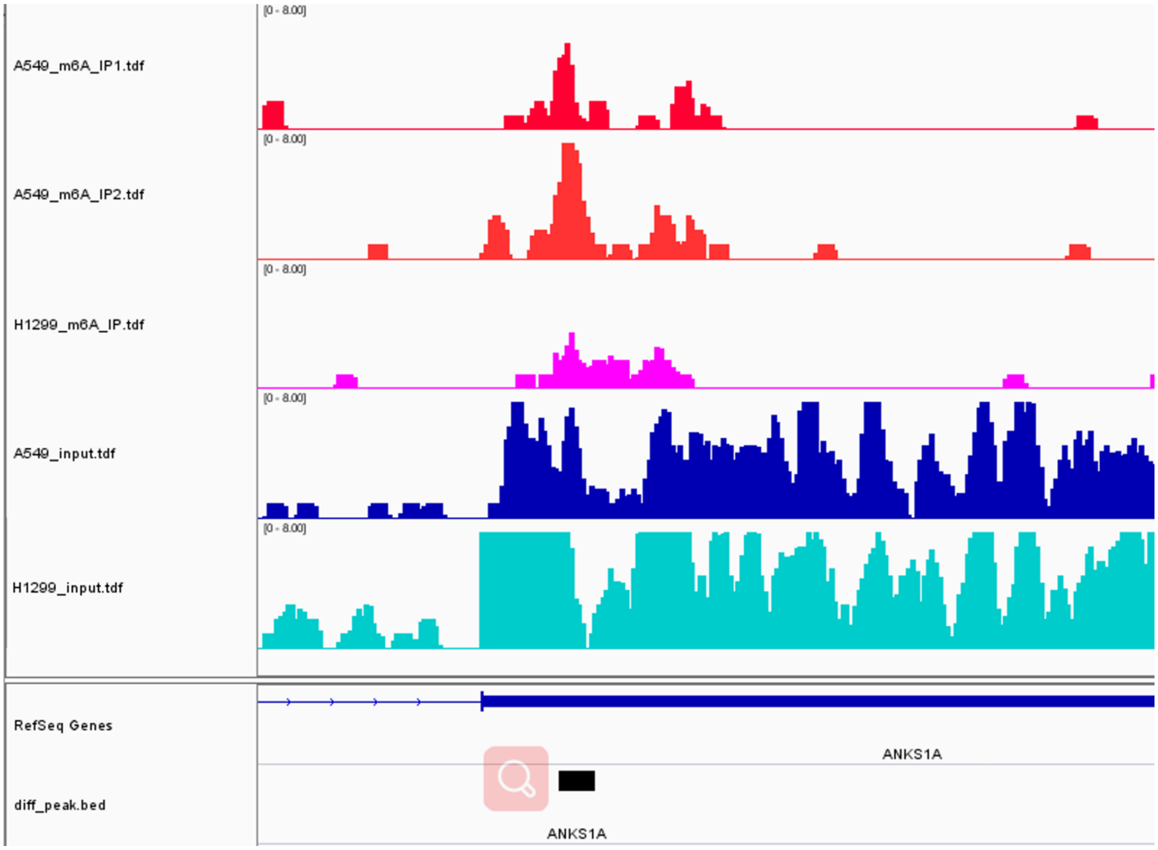

Figure S1. The methylation and expression status of ANKS1A under Non-Small Cell Lung Cancer (A549 cell line) and the normal control (H1299 cell line). The expression fold change of this gene between NSCLC and control condition is -0.798; the methylation level fold change is 1.43.

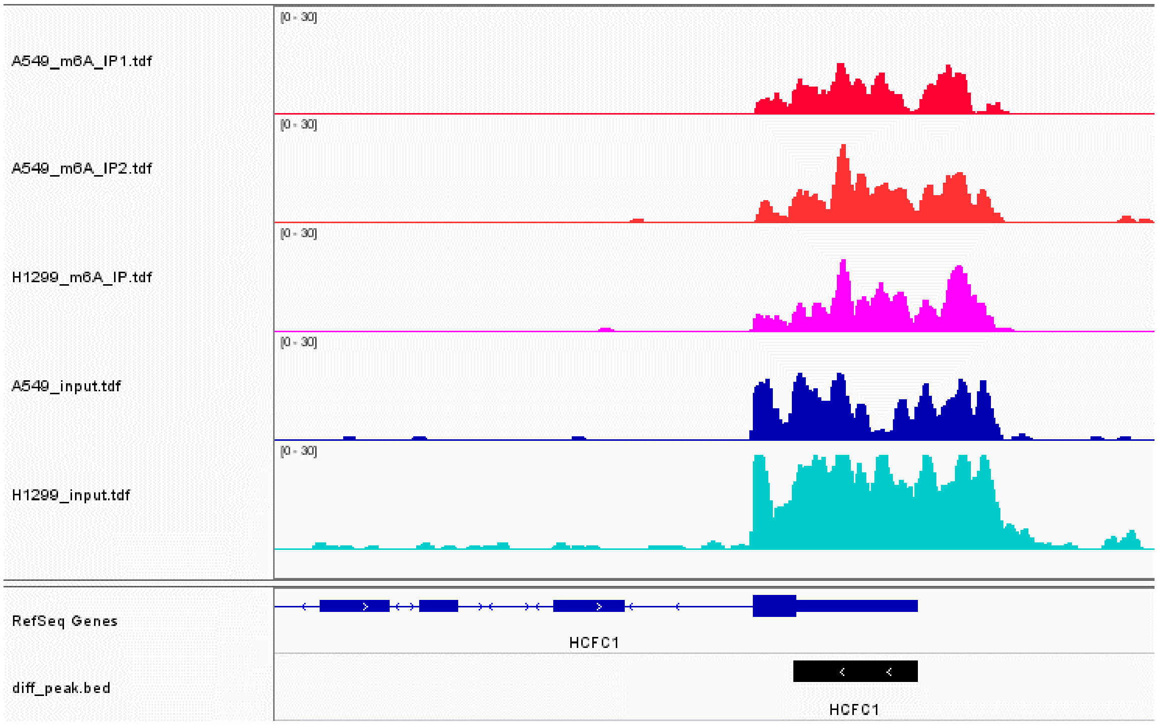

Figure S2. The methylation and expression status of HCKC1 under Non-Small Cell Lung Cancer (A549 cell line) and the normal control (H1299 cell line). The expression fold change of this gene between NSCLC and control condition is -0.728; the methylation level fold change is 0.877.

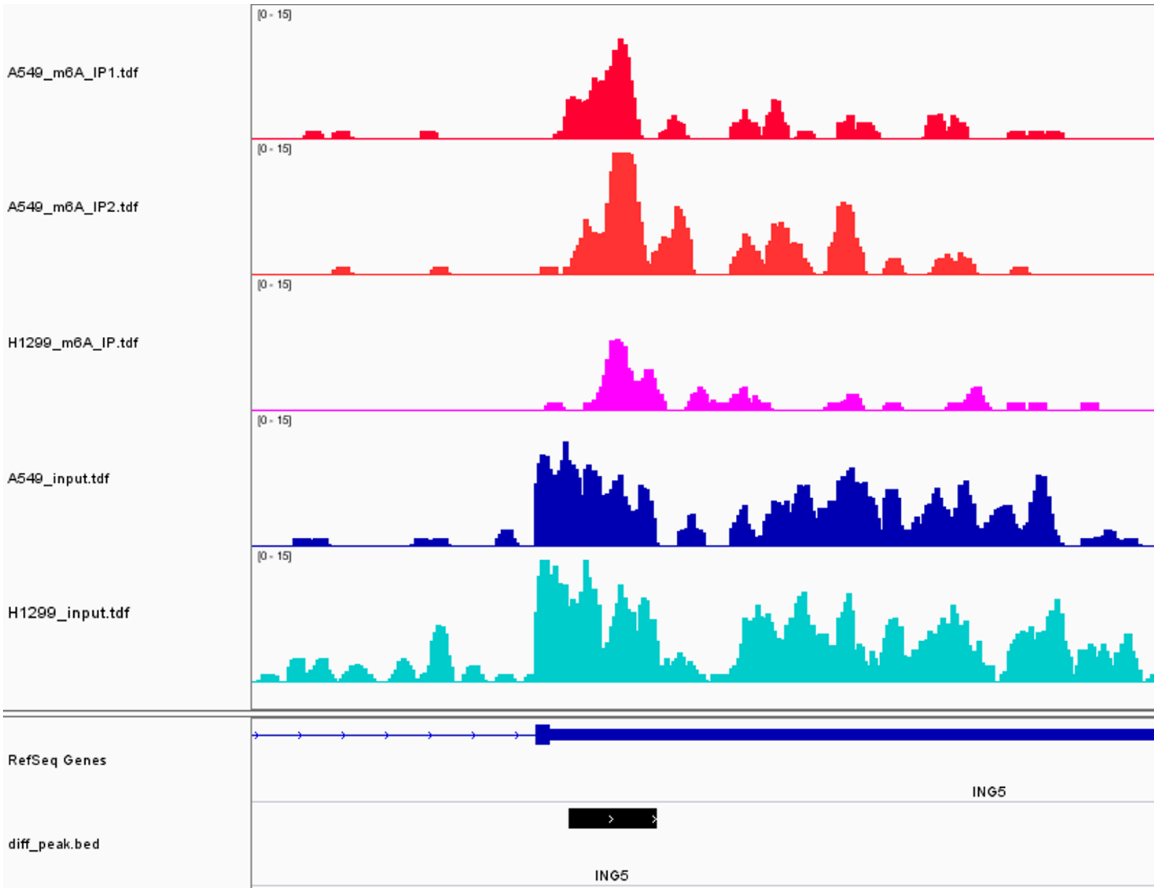

Figure S3. The methylation and expression status of ING5 under Non-Small Cell Lung Cancer (A549 cell line) and the normal control (H1299 cell line). The expression fold change of this gene between NSCLC and control condition is 0.073; the methylation level fold change is 0.762.

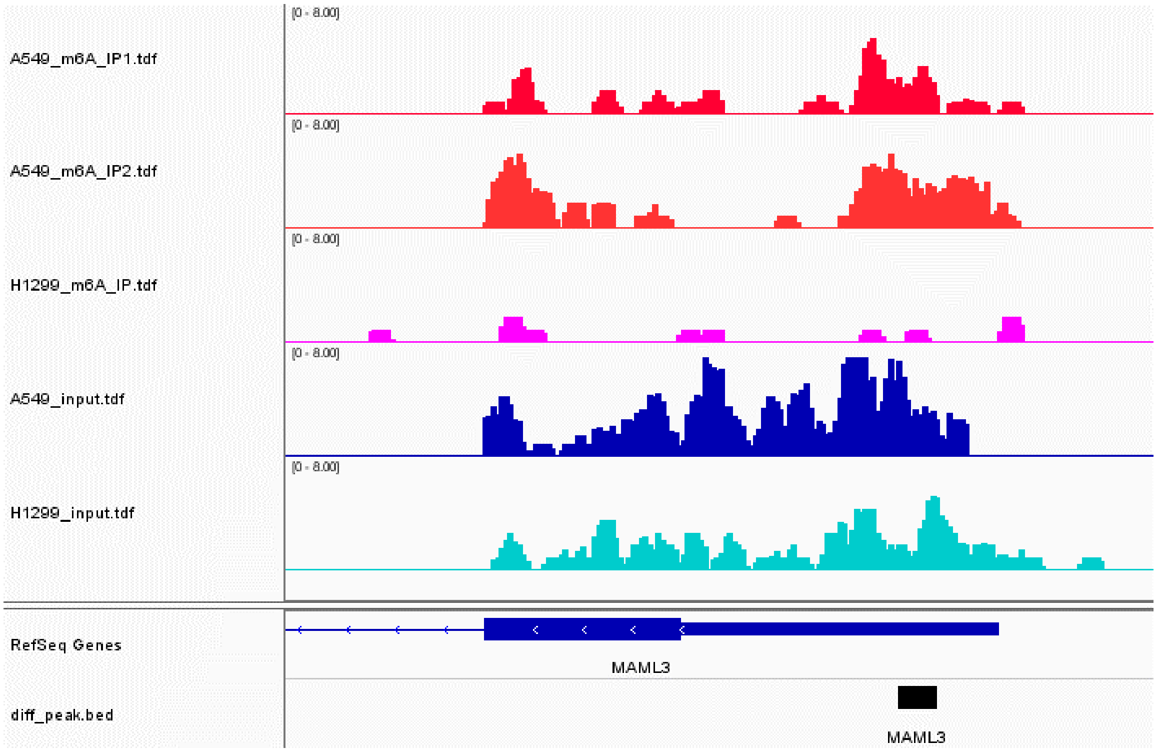

Figure S4. The methylation and expression status of MAML3 under Non-Small Cell Lung Cancer (A549 cell line) and the normal control (H1299 cell line). The expression fold change of this gene between NSCLC and control condition is 2.22; the methylation level fold change is 3.81.

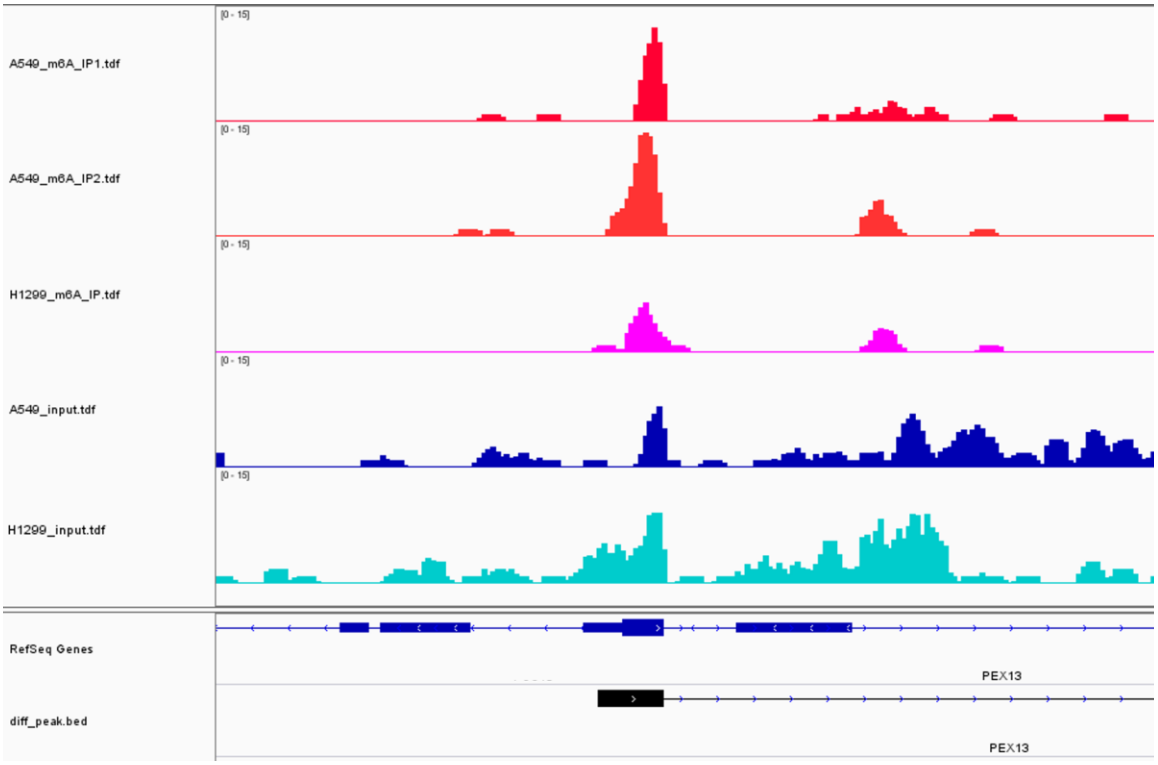

Figure S5. The methylation and expression status of PEX13 under Non-Small Cell Lung Cancer (A549 cell line) and the normal control (H1299 cell line). The expression fold change of this gene between NSCLC and control condition is 1.140; the methylation level fold change for circled site by red is 1.52.

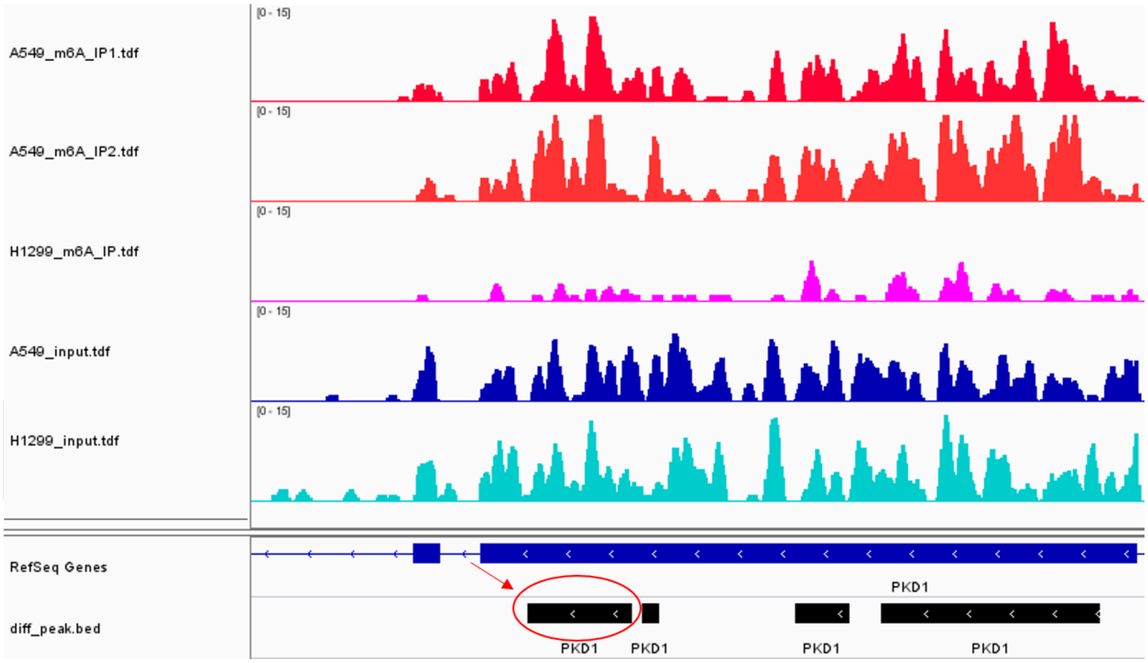

Figure S6. The methylation and expression status of PKD1 under Non-Small Cell Lung Cancer (A549 cell line) and the normal control (H1299 cell line). The expression fold change of this gene between NSCLC and control condition is 1.512; the methylation level fold change for circled site by red is 4.27.

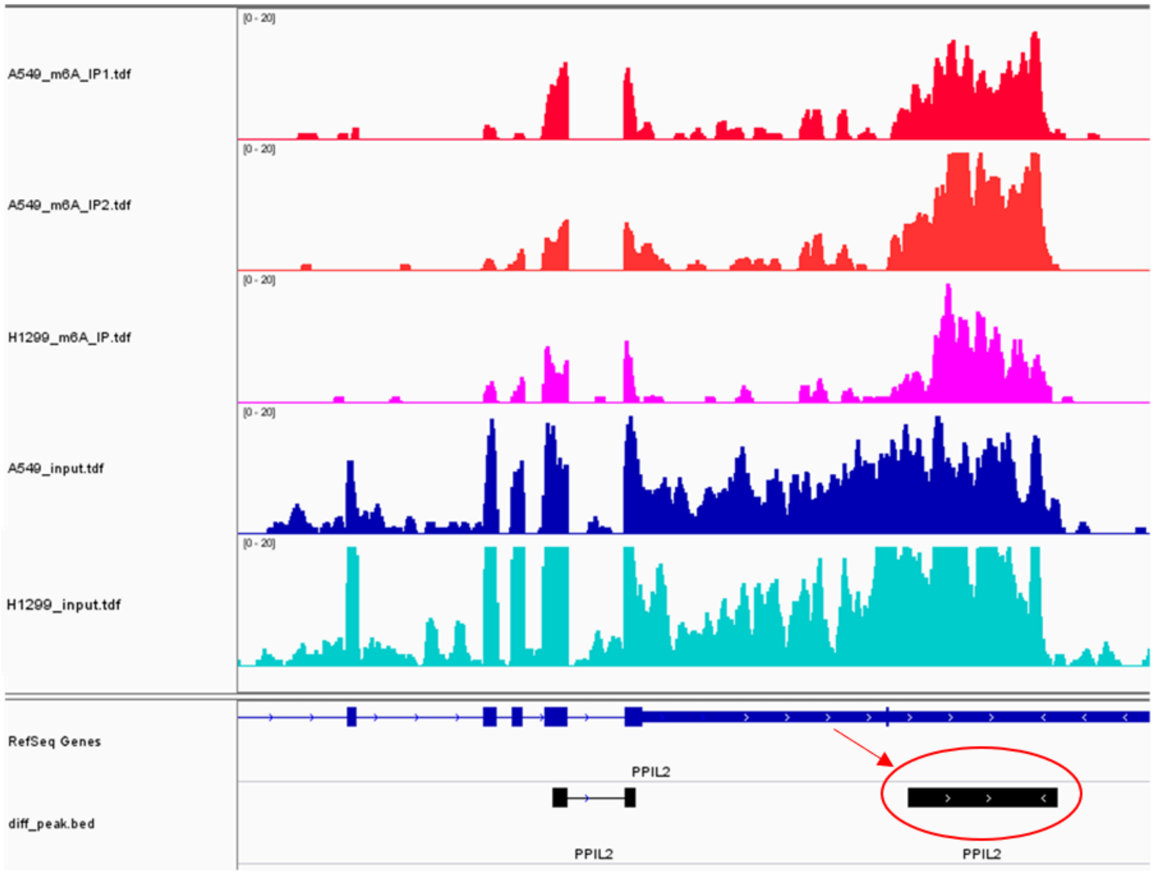

Figure S7. The methylation and expression status of PPIL2 under Non-Small Cell Lung Cancer (A549 cell line) and the normal control (H1299 cell line). The expression fold change of this gene between NSCLC and control condition is -0.791; the methylation level fold change for circled site by red is 0.913.

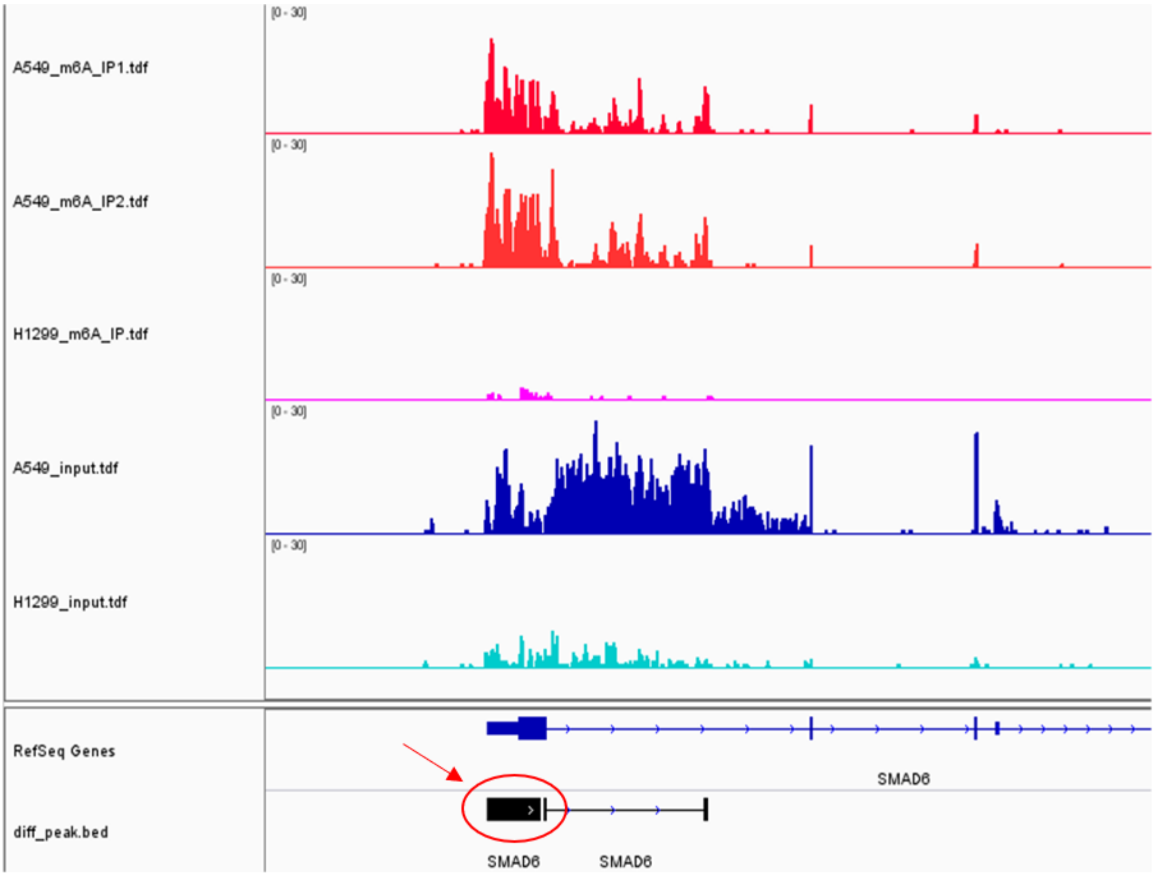

Figure S8. The methylation and expression status of SMAD6 under Non-Small Cell Lung Cancer (A549 cell line) and the normal control (H1299 cell line). The expression fold change of this gene between NSCLC and control condition is 5.244; the methylation level fold change for circled site by red is 4.12.

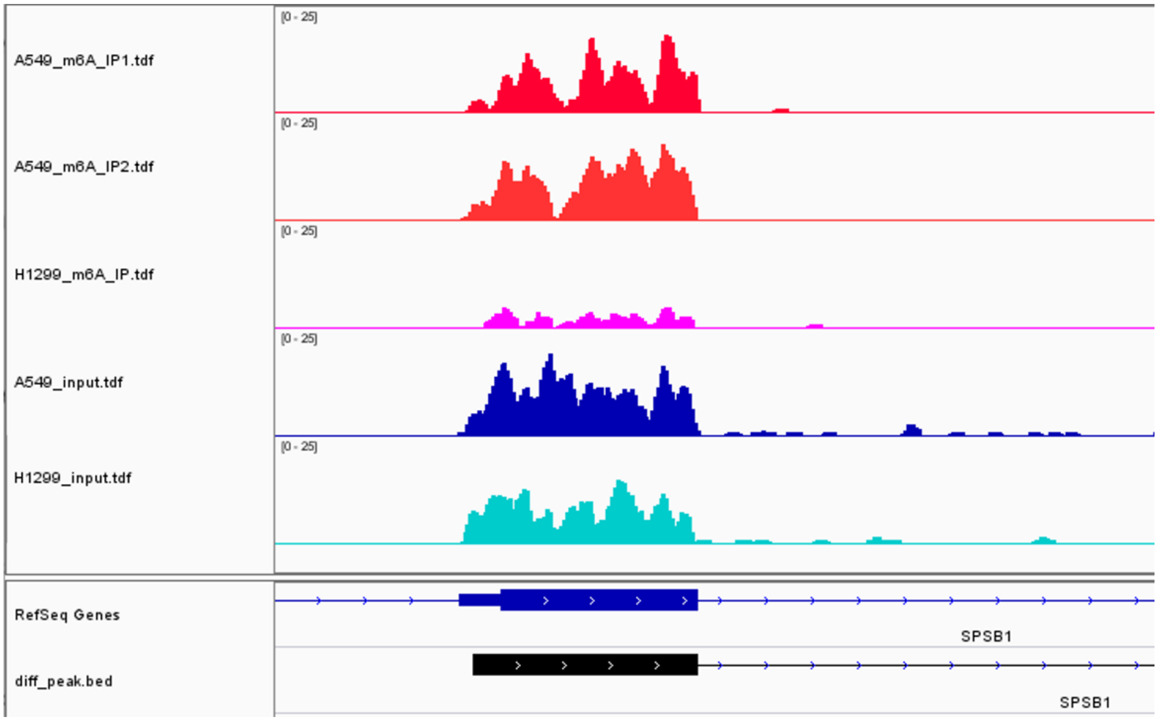

Figure S9. The methylation and expression status of SPSB1 under Non-Small Cell Lung Cancer (A549 cell line) and the normal control (H1299 cell line). The expression fold change of this gene between NSCLC and control condition is 1.888; the methylation level fold change is 2.43.
